# GRIT Score Does Not Correlate With *Quick*DASH Scores in Patients Who Undergo Carpometacarpal Arthroplasty for Thumb Carpometacarpal Arthritis

**DOI:** 10.1016/j.jhsg.2026.101163

**Published:** 2026-10-17

**Authors:** Madeleine G. Wagner, Kimberly S. Khouri, Olivia C. Means, Monica M. Shoji, Carl M. Harper, Tamara D. Rozental

**Affiliations:** ∗ Division of Hand and Upper Extremity Surgery, Department of Orthopedics, Harvard Medical School, Beth Israel Deaconess Medical Center, Boston, MA

**Keywords:** GRIT, Outcomes measurement, *Quick*DASH, Value-based health care

## Abstract

**Purpose:**

Surgery requires resilience when recovering from pain, time off work, and therapy. GRIT is defined as “perseverance and passion for long-term goals” and its relationship to outcomes after surgery has not been well studied. The aim of this study was to examine the correlation between GRIT obtained at 1-year follow-up and self-reported upper extremity physical function as measured via the *Quick*DASH questionnaire among patients undergoing thumb carpometacarpal arthroplasty.

**Methods:**

A retrospective chart review was completed for patients who underwent thumb carpometacarpal arthroplasty from 2022 to 2024. Patients completed the eight-question GRIT Scale, a validated measure of passion and perseverance for long-term goals, summarized on a scale of 1 (least GRIT) to 5 (most GRIT). *Quick*DASH scores were collected before surgery and after surgery at 6 weeks, at 3 months, and at 1 year after surgery. Correlations between GRIT Scale and changes in *Quick*DASH scores were assessed using Spearman correlation coefficients.

**Results:**

Eighty patients (54 females and 26 males) were included, with an average age of 65.8 (SD = 7.6). The mean GRIT score was 3.9 (SD = 0.6). *Quick*DASH score was 44.7 ± 16.2 presurgery and 24.5 ± 18.3 at 1 year or more postsurgery. The number of patients with a decrease in *Quick*DASH from baseline ≥12 (the minimal clinically important difference) was 10 (13%) at 6 weeks, 35 (45%) at 3 months, and 54 (68%) at 12 months postoperatively. Spearman correlation analyses demonstrated no significant associations between GRIT and *Quick*DASH scores at individual postoperative time points. However, in an adjusted linear mixed effects model, higher GRIT scores were independently associated with lower *Quick*DASH scores by 4.21 points, when accounting for age, gender, smoking status, hand dominance, and time from surgery.

**Conclusions:**

Higher GRIT scores were associated with statistically lower postoperative *Quick*DASH scores in adjusted analyses; however, the magnitude of this association was below the reported minimal clinically important difference. Future studies are needed to investigate the association between individual differences in character traits and patient outcomes to better align resources and further the ability to deliver individualized, quality health care.

**Type of study/level of evidence:**

Prognostic IV.

Thumb carpometacarpal (CMC) osteoarthritis is one of the most common degenerative conditions affecting the hand and is a leading cause of pain and functional impairment in older patients, especially women.^1^, ^2^, ^3^ When nonsurgical management such as splinting, activity modification, and corticosteroid injection fails to adequately relieve symptoms, CMC arthroplasty is performed to restore thumb motion, reduce pain, and improve daily function.^4^^,^^5^ Although postoperative outcomes are mostly favorable, patients’ recovery trajectories are variable. Additionally, many patients experience prolonged rehabilitation periods and differences in perceived functional improvement.^6^^,^^7^

In recent years, increasing attention has been directed toward psychological and behavioral factors that may influence recovery after orthopedic surgery. Patient-reported outcome measures (PROMs) provide standardized assessments of patients’ perceptions of pain, function, and health status, allowing clinicians to evaluate treatment outcomes from the patient’s perspective. PROMs have allowed physicians to stratify which patients may or may not benefit from varying treatment plans.^8^ Surgeons must consider inherent patient factors and individual traits that may affect a patient’s PROM results to help deliver more personalized patient care.

One such factor is grit, defined as sustained perseverance and passion toward long-term goals.^9^ Grittier individuals may be more likely to adhere to rehabilitation exercises, cope with discomfort during recovery, and maintain motivation over time.^10^ Other studies within orthopedic surgery suggest that resilience-related traits can influence postoperative satisfaction and functional outcomes.^11^ However, grit is an important characteristic that has yet to be robustly studied in hand surgery. Previous orthopedic research examining the relationship between the GRIT Scale and PROMs functional outcomes in individuals undergoing open reduction internal fixation for distal radius fractures found no significant correlation between these variables.^12^ Given that thumb CMC arthroplasty is performed for a chronic degenerative condition rather than an acute traumatic injury, the influence of psychological traits such as GRIT on postoperative recovery may differ. Therefore, evaluating whether GRIT generalizes patients undergoing elective procedures such as CMC arthroplasty, in which expectations, recovery timelines, and self-directed therapy may differ, has not yet been established.

Therefore, the purpose of this study was to evaluate the association between GRIT and self-reported physical function in patients undergoing CMC arthroplasty. Identifying an association between GRIT and postoperative outcomes is not intended to direct different surgical treatments, but rather to improve preoperative patient counseling, identify patients who may benefit from additional postoperative support or rehabilitation resources, and inform future investigations of psychosocial factors that influence recovery. We hypothesized that higher GRIT scores would be associated with lower (better) 1-year postoperative *Quick*DASH scores following CMC arthroplasty.

## Materials and Methods

We conducted a retrospective review from a single institution’s continuous database of patients who underwent thumb CMC arthroplasty in the form of ligament reconstruction and tendon interposition from January 2022 to December 2024 with a minimum of 1 year of clinical follow-up. All patients presented to one of three fellowship-trained hand surgeons at our institution. The *Quick*DASH questionnaire, a validated upper extremity PROM, is routinely completed by all patients at each visit, with patients typically being seen at 6 weeks, 3 months, and 1 year postsurgery. Postsurgical patients who underwent thumb CMC arthroplasty with complete follow-up to or extending past 1 year were asked to additionally complete the eight-question short GRIT scale. The GRIT scale is a validated measure of passion and perseverance for long-term goals summarized on a scale of 1 (least GRIT) to 5 (most GRIT; Appendix 1, available online on the Journal’s website at https://www.jhsgo.org).^9^ Of note, the GRIT Scale was designed and validated to assess differences between individuals and not within individuals over time, and thus GRIT Scale scores were collected only once at the 1-year follow-up.^13^

Inclusion criteria consisted of patients aged 18 years or older, with thumb CMC arthroplasty performed between January 2022 and December 2024. In total, 114 patients had a CMC arthroplasty during this time and were screened consecutively for inclusion. Twenty-five patients declined participation in the GRIT survey, and 9 patients did not complete 12 months of follow-up. After excluding these 34 patients, there were 80 eligible patients for review. Additional patient characteristics collected include age, gender, race, ethnicity, insurance status, side of injury, and hand dominance. Data were collected using Research Electronic Data Capture (REDCap) tools hosted at our institution.

### Statistical analysis

Statistical power calculations, performed before the study initiation, showed that we needed complete data from 80 patients to achieve at least 80% power at the two-sided alpha level of 0.05 to detect a correlation as low as 0.33 (in absolute value). We used descriptive statistics to summarize the participant characteristics (mean, standard deviation [SD], and median, interquartile range [IQR] for continuous variables, frequencies for categorical variables).

After descriptive statistics were generated, we calculated the Spearman correlation between the GRIT score and *Quick*DASH at each time point and change from baseline; the corresponding 95% confidence intervals were reported to emphasize the magnitude and precision of the observed associations. We used a paired-samples t-test to assess the change in *Quick*DASH score at each time point after surgery (6 weeks, 3 months, 1 year); the normality assumption was evaluated through graphical methods and the Shapiro–Wilk test, with no evidence of a significant violation. To account for multiple testing, *P* values were adjusted using the Bonferroni correction. Furthermore, we calculated the Spearman correlation to evaluate the correlation between GRIT score and change in *Quick*DASH at each time point. We estimated a linear mixed effects model (LMEM) to assess the relationship between GRIT and *Quick*DASH scores, adjusting for age, gender, smoking status, hand dominance, and time from surgery, and accounting for the repeated measures. Model assumptions pertaining to the LMEM were verified by graphical inspection of the diagnostic plots. PROC MIXED from the SAS software (version 9.4) was used to fit the LMEM, with the degrees of freedom estimated using the Kenward–Roger approximation.^14^ Several covariance structures were considered for modeling the repeated measures, including unstructured, compound symmetry, autoregressive, Toeplitz, and antedependent. Given the sample size constraints, unequal visit spacing, and based on the model fit based on the Bayesian information criterion, an antedependent covariance structure was chosen for modeling the repeated data. Covariates were included in the model based on clinical relevance. We created graphical summaries using the R software to visualize the proportion of patients who achieved a clinically meaningful improvement in upper extremity function based on the minimal clinically important difference (MCID) for *Quick*DASH, which has been reported to correspond to a decrease of 12 points.^15^^,^^16^ Statistical significance was set at the two-sided alpha of 0.05.

## Results

The majority of patients were women (n = 54, 68%), and the average age of patients in this cohort was 65.8 (SD = 7.6). Additionally, most patients were White (n = 77, 96%) and Non-Hispanic (n = 76, 95%; Table 1). Approximately half of the patients included had surgery on their dominant hand.

**Table 1:** Patient Demographics: Breakdown of Gender, Race, Ethnicity, Insurance Status, Hand Dominance, and Relevant Comorbidities

Variable	Frequency (%)
Age∗	65.8 ± 7.6
Gender	
Male	26 (32)
Female	54 (68)
Race	
White	77 (96)
Black	3 (4)
Ethnicity	
Hispanic	4 (5)
Non-Hispanic	76 (95)
Type 2 diabetes	
Yes	10 (12)
No	70 (88)
Smoking status	
Current	5 (6)
Former	20 (25)
Never	55 (69)
Dominant hand†	
Right	71 (89)
Left	9 (11)
Side of surgery	
Right	40 (50)
Left	40 (50)
Primary insurance	
Medicare	38 (48)
Medicaid	2 (2)
Commercial	40 (50)

∗ Statistics are presented as mean ± standard deviation.

† For 39 patients (48.8%), the operated hand was the dominant one.

Across the total patient cohort, the average GRIT Scale score was 3.9 (SD = 0.6). The average *Quick*DASH scores before surgery, 6 weeks after surgery, 3 months after surgery, and 1 year after surgery were 44.7 (SD = 16.2), 58.5 (SD = 17.1), 35.8 (SD = 16.7), and 24.5 (SD = 18.3), respectively (Table 2). *Quick*DASH increased by 13.7 points (SD = 23.1) at 6 weeks after surgery and decreased by 8.7 (SD = 22.5) and 19.9 (SD = 19.9) at 3 months and 1 year, respectively (Table 2). Sixty-eight percent of the patients met the threshold for a clinically meaningful decrease in *Quick*DASH at 1 year (Figure 1). The correlation between GRIT and QuickDASH (and its change from baseline) was weak (<0.3 in absolute value) and not statistically significant (*P* > .05) for all the time points (Table 3). The results of the linear mixed effects model, which was used to estimate the effect of GRIT on *Quick*DASH, adjusting for age, gender, smoking status, hand dominance, and time from surgery, are summarized in Table 4. Age and gender did not have a significant association with the *Quick*DASH score. The model indicated an average decrease of 4.21 points (95% CI, –8.40 to –0.02) in *Quick*DASH for each increase in GRIT score by 1 point (measured on a 1–5 scale). Furthermore, there was no significant interaction effect between GRIT score and time from surgery (not shown in the table), which suggests no evidence that GRIT influenced the upper limb function improvement trajectory.

**Table 2:** *Quick*DASH Scores by Timepoint: Summary of *Quick*DASH Scores and Their Changes Recorded at Preoperative Visit, 6-Week Visit, 3-Month Visit, and 1-Year Postoperative Visit

*Quick*DASH in Relation to Time of Surgery	N	Mean ± SD	Change From Baseline	Confidence Interval for Change From Baseline†	% of Patients Having a Decrease in *Quick*DASH Greater Than or Equal to 12 (N of Patients With Meaningful Change/No. of Patients‡ (%))
Preoperative	79	44.7 ± 16.2	NA		
6 weeks	78	58.5 ± 17.1	13.7 ± 23.1∗	(8.00 to 19.42)	10/77 (13)
3 months	79	35.8 ± 16.7	–8.7 ± 22.5∗	(–14.26 to –3.22)	35/78 (45)
1 year	80	24.5 ± 18.3	–19.9 ± 19.9∗	(–24.77 to –15.04)	54/79 (68)

∗ Significant at the 0.05 level, adjusted for multiple comparisons using Bonferroni correction.

† The level for the CI is 0.9833 (1–0.05/3).

‡ Number of patients represents the number of patients for whom the change score could be calculated (no missing *Quick*DASH at baseline or the time point under consideration).

**Figure 1:**
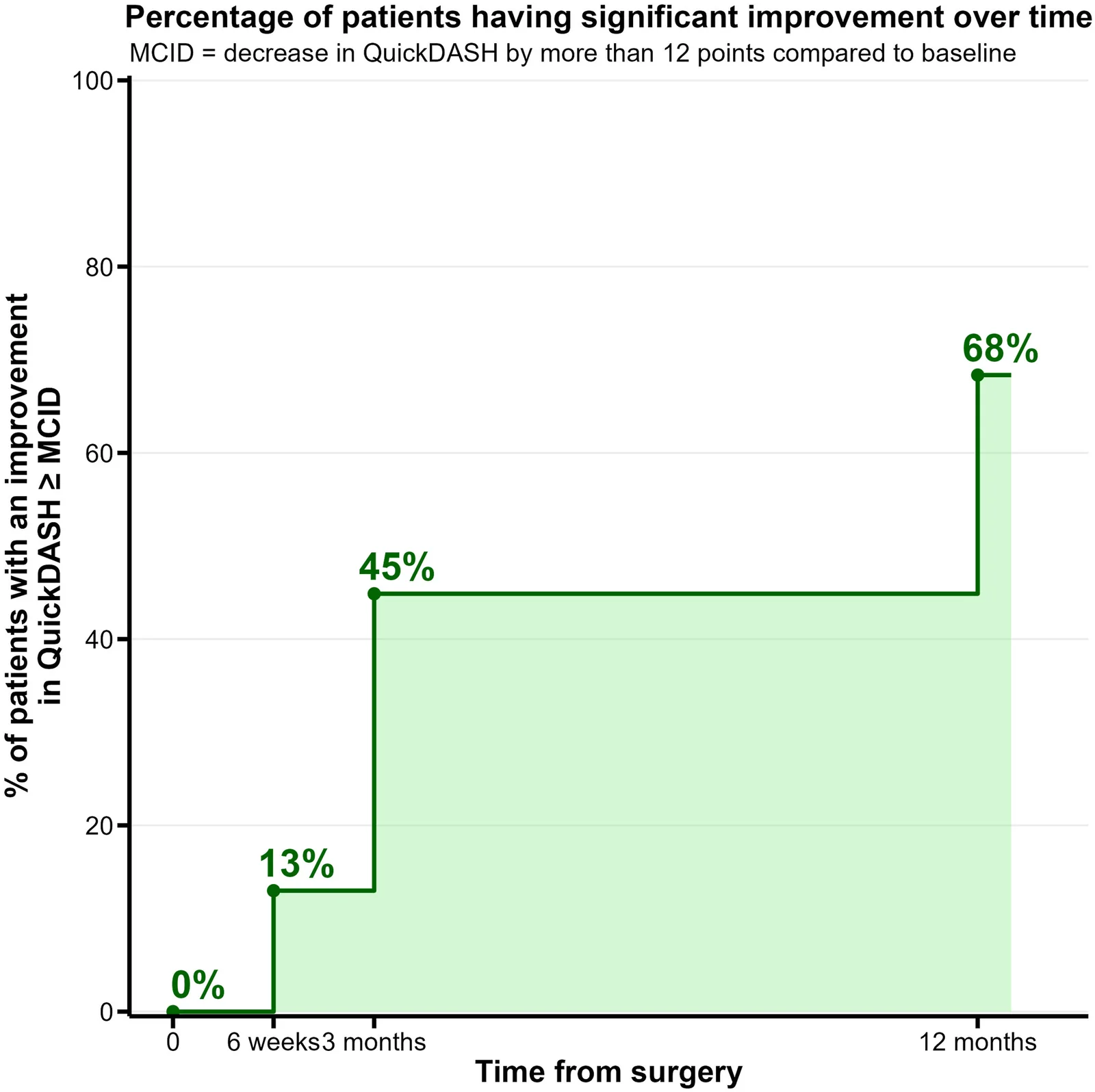
As time from surgery progresses, the proportion of patients having a positive meaningful change in QuickDASH (decrease by more than 12 points, corresponding to the MCID) increases from 13% at 6 weeks to 45% at 3 months to 68% at 1-year postsurgery.

**Table 3:** Correlation Between *Quick*DASH and GRIT Scale Scores

*Quick*DASH in Relation to Time of Surgery	Correlation With Grit Score Spearman ρ (95% CI)	Correlation Between Grit Score and QuickDASH Change Spearman ρ (95% CI)
Preoperative	–0.13 (–0.36 to 0.12)	
6 weeks	–0.07 (–0.29 to 0.16)	0.04 (–0.18 to 0.27)
3 months	–0.12 (–0.33 to 0.10)	0.00 (–0.22 to 0.22)
1 year	–0.02 (–0.24 to 0.20)	0.06 (–0.16 to 0.28)

Note: Spearman correlation coefficient and the corresponding 95% CI evaluating the association between the GRIT score and *Quick*DASH scores measured at each time point (preoperative, 6 weeks, 3 months, and 1 year postoperative) as well as the changes in the *Quick*DASH scores.

**Table 4:** The Results of the Linear Mixed Effects Model

Effect	B Estimate	SE	95% CI (LL to UL)
Intercept	63.8	12.62	(38.77 to 88.83)
Grit score	–4.21	2.11	(–8.40 to –0.02)
Age	0.00	0.16	(–0.31 to 0.31)
Gender∗			
Male	–1.22	2.53	(–6.24 to 3.79)
Smoking†			
Current or former	–6.84	2.5	(–11.81 to –1.88)
Injured hand is the dominant‡			
Yes	0.09	2.34	(–4.55 to 4.73)
Time from surgery§			
6 weeks	13.99	2.58	(8.85 to 19.13)
3 months	–9.2	2.6	(–14.34 to –4.06)
12 months	–20.22	2.72	(–25.60 to –14.84)

Note: This model evaluates the association between the GRIT score and *Quick*DASH, adjusted for relevant clinical covariates. B estimate represents the effect on *Quick*DASH for each corresponding predictor.

LL, lower limit; SE, standard error; UL, upper limit.

∗ Reference category is female.

† Reference category is never smoker.

‡ Reference category is no.

§ Reference category is preoperative.

## Discussion

The focus on patient-specific quality of care and individualized approach to treatment options has heightened the importance of PROMs. To improve clinical outcomes, physicians have to make decisions about the treatment plan while accounting for individual differences in character traits. Previous studies have examined factors associated with hand patients’ self-reported pain and function, including anxiety, depression, and pain catastrophizing, finding a correlation between self-reported anxiety levels and PROMs.^17^ Given the structured and often demanding nature of postoperative hand therapy, GRIT has been considered a potentially relevant factor in orthopedic recovery. However, the degree to which this psychological trait influences functional outcomes after hand surgery remains unclear. Previous research has reported an association between GRIT and improved performance and retention across multiple high-demand environments, including educational settings, military training programs, and surgical residency.^10^

The past literature demonstrates higher preoperative *Quick*DASH scores with a subsequent decrease in *Quick*DASH scores further after surgery for patients undergoing thumb CMC arthroplasty. Our results follow this general pattern, with a decrease in *Quick*DASH compared with the preoperative score by the 1-year follow-up visit.^18^ We found an increase in patients reaching an MCID further out from their surgery, aligning with the previous literature.^19^ We did not identify a significant correlation between GRIT and *Quick*DASH scores at any time point. On the other hand, the linear mixed effects model results suggest that there is a negative relationship between GRIT and *Quick*DASH scores (for each increase in GRIT by 1 point, the *Quick*DASH decreases on average by 4.21 points; *P* = .049), while adjusting for relevant clinical factors. Although this association reached statistical significance, its magnitude was modest. A 1-point increase in GRIT was associated with an average 4.21-point decrease in *Quick*DASH score, which is well below the reported minimal clinically important difference of 12 points. Thus, although GRIT may contribute to variability in postoperative patient-reported outcomes, the observed effect is unlikely to be clinically meaningful for individual patients. Smoking was also independently associated with postoperative *Quick*DASH scores in the adjusted analysis. This finding is inconsistent with prior orthopedic literature demonstrating that smoking is associated with less favorable postoperative recovery and patient-reported outcomes. Notably, our results parallel those seen in patients undergoing open reduction internal fixation for distal radius fractures, in whom GRIT was not associated with self-reported functional outcomes.^12^

Although this study focused solely on thumb CMC arthroplasty, GRIT may contribute to improved PROMs after surgeries requiring longer, more intensive postoperative therapy protocols, such as replants or revascularization procedures. Although we hypothesize that increased GRIT likely leads to increased adherence to postoperative care, including hand therapy, this has not yet been demonstrated for surgical procedures. However, characteristics such as GRIT could influence engagement with occupational therapy or adherence to postoperative recovery protocols. Similar to our results, one study in the orthopedic literature comparing GRIT score and patient outcomes after total joint arthroplasty did not demonstrate a significant improvement in outcomes.^20^ Additional research is needed to assess the role of GRIT in a range of hand and upper extremity procedures and in patients’ ability to adhere to postoperative regimens. The ability to pinpoint intrinsic patient factors that play an important role in the recovery process and ultimate outcomes is essential for improvement of patient outcomes.

There are several limitations to our study. First, our data came from a single academic medical center, and most included patients were White. Thus, the generalizability of our findings may be limited. Second, we only evaluated the correlation of the GRIT Scale to one PROM (ie, *Quick*DASH). Although the *Quick*DASH is a commonly used upper extremity PROM, we recognize there is a multitude of alternative PROMs, such as the Patient-Rated Wrist Evaluation, the Michigan Hand Questionnaire, the Patient-Reported Outcomes Measurement Information System Upper Extremity, and the Oxford Elbow Score. However, prior studies have shown that the *Quick*DASH strongly correlates with some of the previously noted PROMs.^12^^,^^16^^,^^17^ Additional patient-reported outcome measures evaluating complementary domains such as pain, satisfaction, or hand-specific function may provide a more comprehensive assessment of postoperative recovery. Although GRIT is a relatively stable personality trait and was designed to assess differences between individuals rather than changes over time, patients’ experiences during the postoperative recovery and rehabilitation process may have influenced their responses, introducing the potential for reverse causation or response bias. Lastly, we excluded patients with incomplete follow-up, and it is possible that those who were lost to follow-up were less engaged with rehabilitation and had lower GRIT.^17^^,^^21^, ^22^, ^23^

The GRIT Scale is one of many potential avenues for investigating patient characteristics that may play a role in surgical recovery. Although GRIT, when measured in the manner of this study, did not provide sufficient clinical value to stratify functional recovery after CMC arthroplasty, future studies should be prospective, include preoperative GRIT assessment, and evaluate therapy adherence, pain, satisfaction, and health care utilization. Future prospective studies should evaluate whether behavioral factors, including adherence to postoperative rehabilitation protocols, mediate the relationship between psychological characteristics such as GRIT and postoperative recovery. Understanding these mechanisms may help determine whether GRIT has practical value as a prognostic marker or whether other modifiable psychosocial factors better explain differences in patient-reported outcomes. It is also valuable to look for other patient characteristics that can be examined as mechanisms to individualize patient care and to create more effective recovery protocols.

## Ethics declaration

Verbal informed consent to take part in the study and to publish the article was obtained from all participants or their legal representatives. The study protocol specified a verbal prospective consent process. Potential participants were provided with a study summary sheet and discussed the study with a member of the research team before enrollment. Verbal consent was documented by the study team, including the participant’s name, the individual obtaining consent, and the date consent was obtained. The study was approved by the Beth Israel Deaconess Medical Center Institutional Review Board. The privacy rights of participants have been observed.

This study was performed in compliance with relevant laws, regulatory frameworks and guidelines where the research took place. This study was conducted in accordance with 45 CFR parts 160 and 164, subparts A and E, for the purposes of “health care operations” or “research” as those terms are defined at 45 CFR 164.501 or for “public health activities and purposes” as described under 45 CFR 164.512(b). This study was approved by the Beth Israel Deaconess Medical Center. (Approval No. 2022P000922 Version:7)

## Conflicts of Interest

No benefits in any form have been received or will be received related directly to this article.
