# Supplementary material for: GRIT Score Does Not Correlate With QuickDASH Scores in Patients Who Undergo Carpometacarpal Arthroplasty for Thumb Carpometacarpal Arthritis
Source: J Hand Surg Glob Online. 2026 Oct 17;9(1):101163. doi: 10.1016/j.jhsg.2026.101163 (PMC13696140; doi:10.1016/j.jhsg.2026.101163)
Supplement: Supplementary file 1 [file mmc1.pdf]

Short Grit Scale

Directions for taking the Grit Scale: Here are a number of statements that may or may not apply to you. For the most accurate score, when responding, think of how you compare to most people -- not just the people you know well, but most people in the world. There are no right or wrong answers, so just answer honestly!

1. New ideas and projects sometimes distract me from previous ones.*
   - ❑ Very much like me
   - ❑ Mostly like me
   - ❑ Somewhat like me
   - ❑ Not much like me
   - ❑ Not like me at all

2. Setbacks don't discourage me.
   - ❑ Very much like me
   - ❑ Mostly like me
   - ❑ Somewhat like me
   - ❑ Not much like me
   - ❑ Not like me at all

3. I have been obsessed with a certain idea or project for a short time but later lost interest.*
   - ❑ Very much like me
   - ❑ Mostly like me
   - ❑ Somewhat like me
   - ❑ Not much like me
   - ❑ Not like me at all

4. I am a hard worker.
   - ❑ Very much like me
   - ❑ Mostly like me
   - ❑ Somewhat like me
   - ❑ Not much like me
   - ❑ Not like me at all

5. I often set a goal but later choose to pursue a different one.*
   - ❑ Very much like me
   - ❑ Mostly like me
   - ❑ Somewhat like me
   - ❑ Not much like me
   - ❑ Not like me at all

6. I have difficulty maintaining my focus on projects that take more than a few months to complete.*
   - ❑ Very much like me
   - ❑ Mostly like me
   - ❑ Somewhat like me

- ❑ Not much like me
- ❑ Not like me at all

7. I finish whatever I begin.
   - ❑ Very much like me
   - ❑ Mostly like me
   - ❑ Somewhat like me
   - ❑ Not much like me
   - ❑ Not like me at all

8. I am diligent.
   - ❑ Very much like me
   - ❑ Mostly like me
   - ❑ Somewhat like me
   - ❑ Not much like me
   - ❑ Not like me at all

<u>Scoring</u>:

1. For questions 2, 4, 7 and 8 assign the following points:
   5 = Very much like me
   4 = Mostly like me
   3 = Somewhat like me
   2 = Not much like me
   1 = Not like me at all

2. For questions 1, 3, 5 and 6 assign the following points:
   1 = Very much like me
   2 = Mostly like me
   3 = Somewhat like me
   4 = Not much like me
   5 = Not like me at all

Add up all the points and divide by 8. The maximum score on this scale is 5 (extremely gritty), and the lowest score on this scale is 1 (not at all gritty).

Grit Scale citation

Duckworth, A.L, & Quinn, P.D. (2009). Development and validation of the Short Grit Scale (Grit-S). *Journal of Personality Assessment, 91,* 166-174. http://www.sas.upenn.edu/~duckwort/images/Duckworth%20and%20Quinn.pdf

Duckworth, A.L., Peterson, C., Matthews, M.D., & Kelly, D.R. (2007). Grit: Perseverance and passion for long-term goals. *Journal of Personality and Social Psychology, 9*, 1087-1101. http://www.sas.upenn.edu/~duckwort/images/Grit%20JPSP.pdf

© 2007 Angela Duckworth
Do not duplicate or distribute without the consent of the author.
